# Association between ventricular arrhythmia (premature ventricular contractions burden and nonsustained ventricular tachycardia) and cardiovascular events in patients without structural heart disease

**DOI:** 10.1002/joa3.13203

**Published:** 2024-12-15

**Authors:** Sho Ogiso, Takuto Arita, Shinya Suzuki, Naomi Hirota, Naoharu Yagi, Takayuki Otsuka, Mikio Kishi, Hiroto Kano, Shunsuke Matsuno, Yuko Kato, Tokuhisa Uejima, Yuji Oikawa, Junya Ako, Junji Yajima, Takeshi Yamashita

**Affiliations:** ^1^ Department of Cardiovascular Medicine The Cardiovascular Institute Tokyo Japan; ^2^ Department of Cardiovascular Medicine Kitasato University School of Medicine Kanagawa Japan

**Keywords:** 24‐h Holter monitoring, apparently normal heart, heart failure hospitalization, nonsustained ventricular tachycardia, premature ventricular contractions

## Abstract

**Background:**

Premature ventricular contractions (PVCs) and nonsustained ventricular tachycardia (NSVT) are common arrhythmias in cardiovascular clinical settings. However, the clinical significance of PVCs and NSVT in the absence of structural heart disease has not yet been fully elucidated. This study aimed to evaluate the association between PVCs, NSVT, and clinical outcomes.

**Methods:**

A study population of 26,117 patients was drawn from the Shinken Database established in June 2004. We enrolled 6332 patients without structural heart disease who underwent 24‐h Holter monitoring and were registered up to March 2019. We focused on ventricular arrhythmias and cardiovascular events in patients without structural heart diseases. The study population was divided by the number of baseline PVCs (PVCs: <1000 (*n* = 5507), 1000–9999 (*n* = 531), and 10 000 ≤(*n* = 294)). The study population was also divided according to the presence or absence of NSVT (*n* = 454 and *n* = 5878, respectively).

**Result:**

During the follow‐up period up to 3 years, there were 16 deaths, 24 heart failure‐related hospitalizations, 14 acute coronary syndromes, and 37 embolism events. The frequency of PVCs was not associated with mortality or heart failure. On the other hand, the presence of NSVT was significantly associated with heart failure hospitalization in a multivariate model (hazard ratio: 3.02; 95% CI: 1.03–8.83; *p* = .044).

**Conclusion:**

In patients without structural heart disease, NSVT was associated with a higher risk of heart failure hospitalization. Patients with NSVT but no structural heart diseases require careful follow‐up and management of heart failure risk factors.

## Graphical abstract

The clinical significance of premature ventricular contractions (PVCs) and nonsustained ventricular tachycardia (NSVT) without structural heart disease remains unclear. The presence of NSVT was associated with heart failure hospitalization.

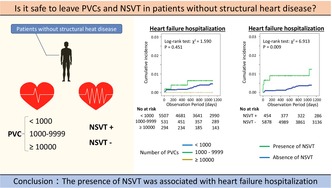

## 1 INTRODUCTION

Premature ventricular contractions (PVCs) are among the most frequently encountered arrhythmias in clinical practice. A higher frequency of PVCs has been linked to an overall increased risk of clinically significant heart failure (HF) and mortality.^1^ However, many patients with frequent PVCs do not develop systolic dysfunction or clinical HF. Little information is available regarding the long‐term follow‐up of patients with apparently normal heart function stratified by PVC burden.^2^

Nonsustained ventricular tachycardia (NSVT) is a frequently encountered arrhythmia in daily clinical settings and is associated with increased mortality in patients with structural heart disease.^3^ A previous study reported that NSVT episodes can be recorded in a normal population and patients without significant structural heart disease.^4^ A previous study reported that the presence of NSVT in patients without structural heart disease is independently associated with death, ischemic stroke, and new‐onset HF.^5^

However, the clinical significance of PVCs and NSVT without structural heart disease is often disregarded, and long‐term clinical outcomes stratified by PVC burden or the presence of NSVT have not been fully described.^4^, ^6^ This study aimed to assess the risk of PVCs and NSVT in patients without apparent structural heart disease.

## 2 METHODS

### 2.1 Study design and setting

This retrospective cohort study used data from the Shinken Database. The Shinken Database has registered all new patients who visited a cardiology‐specialized hospital.^7^, ^8^ The database was established in June 2004, and 26,117 patients were registered until March 2019. The hospital is a specialized cardiovascular hospital in Tokyo, Japan, that receives many referrals from clinics as well as from the local population.

### 2.2 Study participants

We analyzed 6332 patients without structural heart disease who underwent 24‐h Holter monitoring in our single hospital‐based cohort (Shinken Database) between June 2004 and March 2019. We examined the association between ventricular arrhythmias (PVCs burden and NSVT) and cardiovascular events in patients without structural heart disease. The study population was divided by the number of baseline PVCs (PVCs: <1000 (*n* = 5507), 1000–9999 (*n* = 531), and 10 000 ≤(*n* = 294)). The study population was also divided according to the presence or absence of NSVT (*n* = 454 and *n* = 5878, respectively).

### 2.3 Patient follow‐up

Health status, along with the incidence of cardiovascular events and mortality, was recorded from the hospital medical records to form a database. Patients who ceased attending the hospital or were referred to other hospitals were tracked using annual prognosis forms. The data analysis included follow‐up information up to March 2020.

### 2.4 Data collection

Baseline patient characteristics, such as age, gender, height, and weight, were retrieved. Cardiovascular status was assessed using electrocardiography (ECG), echocardiography, 24‐h Holter recording, exercise testing, and blood laboratory data, as determined by the attending physician.

In our institute, the cutoff values for echocardiographic findings were 37≤ left ventricular end‐diastolic diameter ≤54, 20≤ left ventricular end‐systolic diameter ≤36, 7≤ interventricular septal thickness ≤11, 7≤ posterior wall thickness ≤11, 58≤ left ventricular ejection fraction ≤82, and 23≤ left atrium diameter ≤42. Structural heart diseases were diagnosed comprehensively based on patient symptoms and data using ECG, echocardiography, exercise testing, and blood laboratory as determined by the attending physician. Structural heart disease was defined according to the following criteria: Ischemic heart disease was identified by angiography or scintigraphy. Hypertrophic and dilated cardiomyopathies were diagnosed by using echocardiography or cardiac magnetic resonance (CMR) imaging. Valvular heart disease was defined as a moderate or severe stenosis or regurgitation on echocardiography. HF was diagnosed in patients exhibiting symptoms consistent with New York Heart Association (NYHA) functional class II or higher. Given the association between PVCs/NSVT and abnormal echocardiographic findings including LV dysfunction, the presence of unexplained mild global LV dysfunction (50≤ left ventricular ejection fraction <58), diastolic dysfunction and other borderline echocardiographic parameter alone on initial evaluation without symptomatic heart failure (≤NYHA functional class II) were not diagnosed to have apparently structural heart disease.

Cardiovascular risk factors were characterized using specific criteria. For hypertension, the criteria included the use of antihypertensive medication, a systolic blood pressure of 140 mmHg or higher, or a diastolic blood pressure of 90 mmHg or higher. Diabetes mellitus was identified by the use of oral hypoglycemic drugs or insulin or a glycosylated hemoglobin level exceeding 6.5%. Dyslipidemia was defined as the use of statins, low‐density lipoprotein level >140 mg/dL, high‐density lipoprotein level <40 mg/dL, or fasting triglyceride level >150 mg/dL. Chronic kidney disease (CKD) was diagnosed based on an estimated glomerular filtration rate of less than 60 mL/min/1.73 m^2^.

In this study, the number of PVCs was diagnosed based on 24‐h Holter monitor recordings, and NSVT was diagnosed using electrocardiography at the initial visit, which included either 12‐lead surface ECG or 24‐h Holter monitor recordings. NSVT was defined as those who had three or more consecutive PVCs.

### 2.5 Outcomes

In this study, the primary endpoint was defined as the incidence of cardiovascular events, including all‐cause death, acute coronary syndrome, ischemic stroke, systemic embolism, and hospitalization for HF within 3 years from the first visit to our hospital. Incidence of cardiovascular events were also evaluated in subgroups of patients divided by the number of baseline PVCs, and patients divided by the presence or absence of NSVT.

### 2.6 Statistical analysis

Statistical analyses were performed using SPSS version 19.0 (SPSS Inc., Chicago, IL, USA). Statistical significance was set at *p* < .05. Continuous variables were presented as mean ± standard deviation, while categorical variables were displayed as absolute numbers with corresponding percentages. Differences in categorical variables between the groups were evaluated using the chi‐squared test. For continuous variables, differences between groups were assessed using an unpaired *t*‐test. Survival analysis was performed using the Kaplan–Meier method, with the log‐rank test employed for group comparisons. Univariate and multivariate Cox regression analyses were performed to estimate the hazard ratio (HR) for clinical events. In the multivariable models, the number of baseline PVCs, the presence of NSVT, and covariates with a *p* value of less than .10 in the univariable models were included.

## 3 RESULTS

In this study, there were 16 deaths, 24 HF‐related hospitalizations, 14 acute coronary syndromes, and 37 embolism events during the follow‐up period of up to 3 years (median 1095 (IQR: 560–1095) days).

**FIGURE 1:**
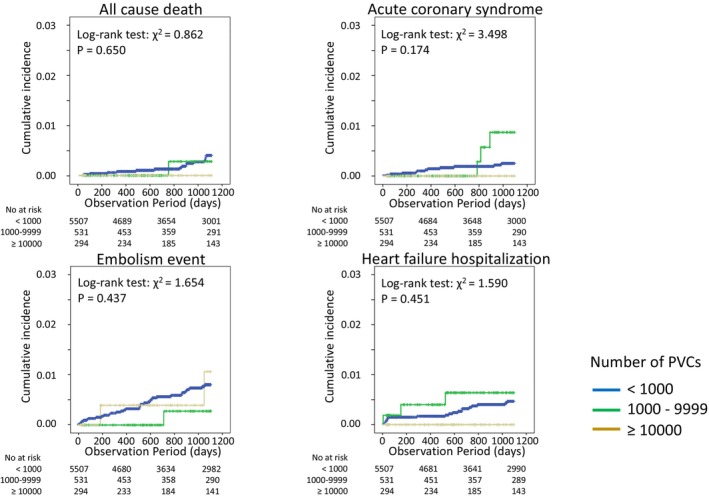
Kaplan–Meier curve for prognosis divided by the PVCs. Kaplan–Meier curves for all‐cause, acute coronary syndrome, ischemic stroke or systemic embolism, and heart failure hospitalization within 3 years based on the number of premature ventricular contractions. PVCs, premature ventricular contractions.

Table 1 shows the characteristics of the study population, which was divided into three groups according to the number of baseline PVCs. In baseline characteristics, patients with a large number of PVCs were significantly younger, taller, and had a lower prevalence of atrial fibrillation (AF) compared to those with a small number of PVCs. They also had more use of class II antiarrhythmic drugs and a higher rate of catheter ablation procedures. The Kaplan–Meier curves for all‐cause death, acute coronary syndrome, ischemic stroke or systemic embolism, and HF hospitalization according to the number of PVCs are shown in Figure 1. The cumulative incidence of clinical events at 3 years among patients with a total of <1000, 1000–9999, and ≥10 000 PVCs were 0.3%, 0.2%, and 0%, respectively, for all‐cause death (log‐rank test: *p* = 0650), 0.2%, 0.6%, and 0%, respectively, for acute coronary syndrome (log‐rank test: *p* = .174), 0.6%, 0.2%, and 0.7%, respectively, for ischemic stroke or systemic embolism (log‐rank test: *p* = .437), and 0.4%, 0.8% and 0% for HF hospitalization (*p* = .451).

**TABLE 1:** Baseline characteristics according to the number of baseline PVCs.

	Number of PVCs (total, *n* = 6332, 100.0%)			*p* value
	0–999 (*n* = 5507)	1000–9999 (*n* = 531)	10,000– (*n* = 294)	
Age (years)	56.0 ± 14.9	55.6 ± 15.2	53.8 ± 16.1	.043
Height (cm)	163.6 ± 9.4	163.9 ± 9.1	165.0 ± 8.7	.030
Weight (kg)	62.1 ± 16.3	62.9 ± 12.7	62.4 ± 13.2	.485
BMI (kg/m^2^)	23.0 ± 5.0	23.3 ± 3.6	22.8 ± 3.5	.379
Male, *n* (%)	3040 (55)	306 (58)	165 (56)	.546
Hypertension, *n* (%)	1467 (27)	157 (30)	74 (25)	.281
Dyslipidemia, *n* (%)	1345 (24)	123 (23)	48 (16)	.006
Diabetes mellitus, *n* (%)	383 (7)	40 (8)	21 (7)	.880
Hyperuricemia, *n* (%)	687 (12)	56 (11)	30 (10)	.242
Chronic kidney disease, *n* (%)	656 (12)	59 (11)	24 (8)	.137
Anemia, *n* (%)	67 (1)	8 (2)	3 (1)	.799
Atrial fibrillation, *n* (%)	1113 (20)	65 (12)	9 (3)	<.001
Antiarrhythmic drugs, *n* (%)	1165 (21)	104 (20)	66 (22)	.588
Class I Antiarrhythmic drugs, *n* (%)	532 (10)	37 (7)	20 (7)	.040
Class II Antiarrhythmic drugs, *n* (%)	506 (9)	62 (12)	48 (16)	<.001
Class III Antiarrhythmic drugs, *n* (%)	35 (1)	3 (1)	1 (0)	.810
Class IV Antiarrhythmic drugs, *n* (%)	381 (7)	20 (4)	11 (4)	.003
Catheter ablation for PVCs/NSVT within 3 years, *n* (%)	1 (0)	5 (1)	16 (5)	<.001

*Note*: Categorical variables were presented as numbers (percentages) and continuous values are presented as mean ± standard deviation.

Abbreviations: BMI, body mass index; NSVT, nonsustained ventricular tachycardia; PVCs, premature ventricular contractions.

**FIGURE 2:**
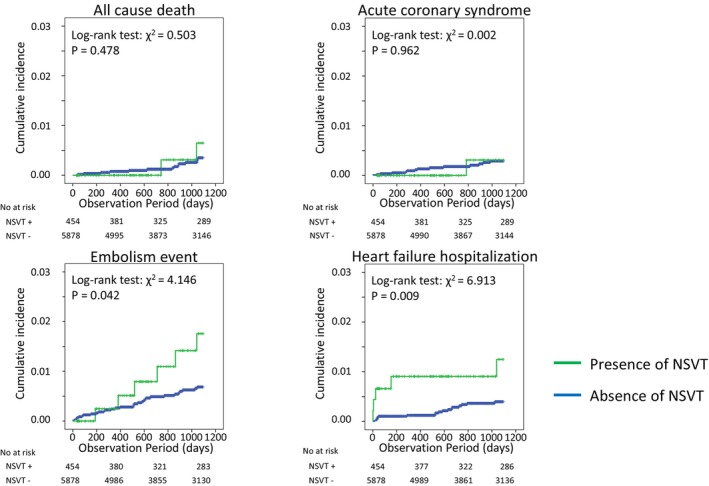
Kaplan–Meier curve for prognosis divided by the NSVT. Kaplan–Meier curves for all‐cause death, acute coronary syndrome, ischemic stroke or systemic embolism, and heart failure hospitalization within 3 years due to the presence of nonsustained ventricular tachycardia. NSVT, nonsustained ventricular tachycardia.

Table 2 summarizes the characteristics of the study population based on the presence of NSVT. Patients with NSVT were significantly older, taller, and comprised a higher proportion of males. Patients with NSVT have a higher prevalence of hypertension, hyperuricemia, CKD, and AF than those without NSVT. Patients with NSVT had more use of Class II antiarrhythmic drugs and higher rates of catheter ablation. None of the patients showed signs of HF (symptoms ≥ NYHA Class II) at baseline. The Kaplan–Meier curves for all‐cause death, acute coronary syndrome, ischemic stroke or systemic embolism, and hospitalization for HF due to the presence of NSVT are shown in Figure 2. The cumulative incidence of clinical events at 3 years in patients with and without NSVT was 0.4% and 0.2% for all‐cause death (*p* = .478), 0.2% and 0.2% for acute coronary syndrome (*p* = 0.962), 1.3% and 0.5% for ischemic stroke or systemic embolism (*p* = .042), and 1.3% and 0.3% for HF hospitalization (log‐rank test: *p* = .009), respectively. In Cox regression analysis, the HRs of the presence of NSVT for the incidence of all‐cause death, acute coronary syndrome, ischemic stroke or systemic embolism, and HF hospitalization were 1.70 (95% CI: 0.39–7.48), 0.95 (95% CI: 0.12–7.28), 2.41 (95% CI 1.01–5.75), and 3.48 (95% CI: 1.29–9.37), respectively, in the univariable models (Table 3 A–D). In multivariate models, the HRs of the presence of NSVT for the incidence of ischemic stroke or systemic embolism and HF hospitalization were 2.09 (95% CI 0.81–5.40) and 3.02 (95% CI: 1.03–8.83) (Table 3 C,D).

**TABLE 2:** Baseline characteristics according to the existence of NSVT.

	NSVT (−) (*n* = 5878)	NSVT (+) (*n* = 454)	*p value*
Age (years)	55.5 ± 15.0	59.9 ± 15.1	<.001
Height (cm)	163.6 ± 9.4	165.0 ± 8.8	.002
Weight (kg)	62.1 ± 16.1	63.4 ± 12.4	.078
BMI (kg/m^2^)	23.0 ± 4.9	23.2 ± 3.4	.514
Male, *n* (%)	3210 (55)	301 (66)	<.001
Hypertension, *n* (%)	1517 (26)	181 (40)	<.001
Dyslipidemia, *n* (%)	1420 (24)	96 (21)	.147
Diabetes mellitus, *n* (%)	414 (7)	30 (7)	.726
Hyperuricemia, *n* (%)	699 (12)	74 (16)	.014
Chronic kidney disease, *n* (%)	661 (11)	78 (17)	.001
Anemia, *n* (%)	73 (1)	5 (1)	.794
Atrial fibrillation, *n* (%)	1081 (18)	106 (23)	.016
Antiarrhythmic drugs, *n* (%)	1178 (20)	157 (35)	<.001
Class I Antiarrhythmic drugs, *n* (%)	542 (9)	47 (10)	.424
Class II Antiarrhythmic drugs, *n* (%)	507 (9)	109 (24)	<.001
Class III Antiarrhythmic drugs, *n* (%)	34 (1)	5 (1)	.296
Class IV Antiarrhythmic drugs, *n* (%)	382 (6)	30 (7)	.928
Catheter ablation for PVCs/NSVT within 3 years, *n* (%)	8 (0)	14 (3)	<.001

*Note*: Categorical variables were presented as numbers (percentages) and continuous values are presented as mean ± standard deviation.

Abbreviations: BMI, body mass index; NSVT, nonsustained ventricular tachycardia; PVCs, premature ventricular contractions.

**TABLE 3:** Univariable and multivariable Cox regression analysis for all‐cause death, acute coronary syndrome, ischemic stroke or systemic embolism, and heart failure hospitalization.

	Univariable model		Multivariable model	
	HR (95% CI)	*p* value	HR (95% CI)	*p* value
A. All‐cause death				
Age (per year)	1.11 (1.06–1.16)	<.001	1.10 (1.04–1.16)	<.001
Gender category (male)	2.30 (0.74–7.14)	.149		
Hypertension	5.65 (1.96–16.27)	.001	3.64 (1.25–10.58)	.018
Dyslipidemia	0.96 (0.31–2.97)	.939		
Diabetes mellitus	2.85 (0.81–10.01)	.102		
Hyperuricemia	2.21 (0.71–6.87)	.169		
Chronic kidney disease	3.17 (1.10–9.13)	.032	1.07 (0.36–3.23)	.903
Anemia	0.05 (0.00–19760919.15)	.765		
Atrial fibrillation	0.83 (0.24–2.90)	.765		
PVC 0–999	reference			
1000–9999	0.68 (0.09–5.17)	.712	0.574 (0.07–4.85)	.611
≥10 000	0.00 (0.00–0.00)	.980	0.00 (0.00–0.00)	.977
NSVT	1.70 (0.39–7.48)	.483	1.46 (0.30–7.00)	.638
B. Acute coronary syndrome				
Age (per year)	1.05 (1.01–1.10)	.022	1.04 (1.00–1.09)	.073
Gender category (male)	4.66 (1.04–20.82)	.044	4.94 (1.07–22.88)	.041
Hypertension	1.99 (0.69–5.74)	.202		
Dyslipidemia	1.18 (0.37–3.76)	.782		
Diabetes mellitus	2.09 (0.47–9.32)	.336		
Hyperuricemia	2.72 (0.85–8.68)	.091	1.31 (0.38–4.52)	.669
Chronic kidney disease	5.37 (1.86–15.48)	.002	3.13 (0.93–10.52)	.065
Anemia	0.05 (0.00–76654468.06)	.780		
Atrial fibrillation	1.03 (0.29–3.71)	.959		
PVC 0–999	reference			
1000–9999	2.80 (0.78–10.05)	.114	3.34 (0.87–12.77)	.078
≥10 000	0.00 (0.00–0.00)	.983	0.00 (0.00–0.00)	.983
NSVT	0.95 (0.12–7.28)	.962	0.44 (0.05–3.75)	.451
C. Ischemic stroke or systemic embolism				
Age (per year)	1.05 (1.02–1.08)	<.001	1.03 (1.00–1.06)	.033
Gender category (male)	1.14 (0.59–2.20)	.698		
Hypertension	2.52 (1.32–4.81)	.005	1.50 (0.77–2.93)	.229
Dyslipidemia	1.09 (0.53–2.25)	.814		
Diabetes mellitus	1.52 (0.54–4.29)	.430		
Hyperuricemia	2.54 (1.23–5.25)	.012	1.57 (0.73–3.35)	.246
Chronic kidney disease	3.05 (1.51–6.18)	.002	1.15 (0.52–2.55)	.728
Anemia	4.67 (1.12–19.41)	.034	4.93 (1.14–21.30)	.033
Atrial fibrillation	6.30 (3.24–12.25)	<.001	4.72 (2.33–9.56)	<.001
PVC 0–999	reference			
1000–9999	0.30 (0.04–2.20)	.237	0.29 (0.04–2.23)	.235
≥10,000	1.17 (0.28–4.89)	.826	1.58 (0.34–7.42)	.560
NSVT	2.41 (1.01–5.75)	.049	2.09 (0.81–5.40)	0.163
D. Heart failure hospitalization				
Age (per year)	1.04 (1.00–1.07)	.028	1.01 (0.98–1.05)	.545
Gender category (male)	0.85 (0.38–1.93)	.701		
Hypertension	2.04 (0.90–4.66)	.089	1.18 (0.50–2.78)	.707
Dyslipidemia	0.48 (0.13–1.51)	.193		
Diabetes mellitus	3.55 (1.32–9.55)	.012	2.46 (0.89–6.82)	.083
Hyperuricemia	1.03 (0.31–3.47)	.961		
Chronic kidney disease	3.17 (1.30–7.70)	.011	1.58 (0.59–4.19)	.362
Anemia	3.68 (0.50–27.33)	.202		
Atrial fibrillation	7.35 (3.12–17.35)	<.001	5.37 (2.20–13.07)	<.001
PVC 0–999	reference			
1000–9999	1.54 (0.46–5.19)	.485	1.31 (0.36–4.82)	.685
≥10 000	0.00 (0.00–0.00)	.976	0.00 (0.00–0.00)	.974
NSVT	3.48 (1.29–9.37)	.014	3.02 (1.03–8.83)	.044

*Note*: In the multivariable model, the category of the number of medications and the covariables with *p* value <.1 in the univariable models forcedly introduced.

Abbreviations: CI, confidence interval; HR, hazard ratio; NSVT, nonsustained ventricular tachycardia; PVC, premature ventricular contraction.

When we focused on five patients who developed HF (Table 4), four of five cases had preserved ejection fraction at the time of HF event (Case no.5 had reduced ejection fraction (EF) (36%) at the time of HF). Three of the five patients hospitalized for HF had concomitant AF. Three of five cases were accompanied with more than seven number of consecutive beats in NSVT, and two of five cases had just three number of consecutive beats in NSVT. The echocardiographic parameters of patients with available data according to baseline PVCs and NSVT are shown in Tables S1 and S2. Regarding the baseline echocardiographic parameters, patients with a large number of PVCs had a significantly larger left ventricular (LV) diameter and lower LVEF. Regarding the baseline echocardiographic parameters, patients with NSVT had significantly thicker walls, larger LV diameters, larger left atrium diameters, higher right ventricular systolic pressures, and lower mitral valve annular early filling tissue Doppler velocity.

**TABLE 4:** Patients with NSVT at baseline who developed heart failure.

Variables	CASE no.				
	1	2	3	4	5
Age (years)	80	58	80	78	66
Gender	Male	Male	Male	Female	Male
Hypertension	No	No	No	Yes	Yes
Dyslipidemia	No	No	No	Yes	No
Diabetes mellitus	Yes	No	No	No	Yes
Hyperuricemia	No	No	No	No	No
Chronic kidney disease	No	No	No	Yes	No
Anemia	No	No	No	No	No
Atrial fibrillation	Yes	No	Yes	No	Yes
Type of heart failure	preserved	preserved	preserved	preserved	reduced
Structural heart disease later identified	No	HCM	No	No	No
Number of consecutive NSVT beats (beats)	3	12	25	7	3
IVST (mm)	9.0	12.0	10.0	10.0	9.0
PWT (mm)	9.0	10.0	9.0	10.0	7.0
LVDd (mm)	46.0	45.0	49.0	49.0	52.0
LVDs (mm)	31.0	24.0	29.0	34.0	43.0
LVEF (%)	61.0	78.0	72.0	58.0	50.0
LAD (mm)	33.0	30.0	34.0	37.0	50.0
RVSP (mmHg)	41	28	44	32	
E (cm/s)	42	24		58	
A (cm/s)	29	42		84	
E/A	1.5	0.6		0.7	
e' (cm/s)				5.2	
E/e' ratio				11.2	

Abbreviations: A, atrial filling velocity; E, early diastolic filling velocity; e', Mitral valve annular early filling tissue Doppler velocity; E/e' ratio, the ratio of diastolic filling velocity divided by average e'; HCM, hypertrophic cardiomyopathy; IVST, interventricular septal thickness; LAD, Left atrium diameter; LVDd, left ventricular end‐diastolic diameter; LVDs, left ventricular end‐systolic diameter; NSVT, nonsustained ventricular tachycardia; LVEF, left ventricular ejection fraction; RVSP, right ventricular systolic pressure; PWT, posterior wall thickness.

## 4 DISCUSSION

### 4.1 Major findings

Overall, the patients without overt structural heart disease had a lower prevalence of cardiovascular events. In our study population, patients with a large number of PVCs were significantly younger than those with fewer PVCs. The frequency of PVCs was not associated with clinical events, including mortality or HF. Notably, patients with a total of ≥10 000 PVCs experienced no death and developed no HF hospitalization. The presence of NSVT was associated with the incidence of ischemic stroke or systemic embolism and was independent of hospitalization due to HF. Patients in the NSVT group who developed HF were relatively old and were mainly hospitalized for HF with a preserved ejection fraction (HFpEF) profile, sometimes accompanied by AF.

### 4.2 Properties of PVCs

Several previous studies have shown that a high burden of PVCs and PVC morphology are associated with clinical outcomes.^9^, ^10^ In a previous study, frequent PVCs can induce cardiac decompensation, and catheter ablation can improve this condition and alleviate related symptoms.^11^ In our study population, the frequency of PVCs (<1000, 1000–9999, and ≥10 000) was not associated with clinical events, which is partly inconsistent with previous reports. Surprisingly, our study had fewer events in a large number of patients with PVCs (no deaths, no acute coronary syndrome events, only two embolism events, and no HF event). The patient characteristics of high‐burden PVCs and medical treatment may explain these results. In this study, Holter ECG was performed not only to detect PVC burden but also to detect all kinds of arrhythmias, including premature atrial contractions and AF, in daily clinical settings. In previous studies, PVCs were associated with old age,^1^, ^12^ which is inconsistent with our results. In our study population, the patients with a large number of PVCs were significantly younger. Frequent PVCs (10%–20% or >20% PVC burden) can be observed in young populations with normal hearts, which have been thought to be benign. In such a population, the PVC burden temporarily declines without the emergence of VT or development of left ventricle dysfunction.^13^

In a previous study, Lin et al. demonstrated that not uniform PVC but multiform PVCs in structurally normal hearts were associated with an increased risk of mortality, hospitalization, and new‐onset HF, independent of other clinical risks.^10^ Although our study did not classify patients based on PVC morphology, PVC burden and morphology may play a significant role in the clinical outcomes of patients with normal hearts.

Furthermore, the effect of medical interventions such as antiarrhythmic drugs and catheter ablation in patients with a large number of PVCs may contribute to a better prognosis.

### 4.3 NSVT and heart failure

In our population, the presence of NSVT was associated with ischemic stroke or systemic embolism and was independently associated with HF hospitalization. The mechanism underlying the relationship between NSVT and HF hospitalization may not be simple.

The characteristics of patients who developed HF in the NSVT group may help consider the relationship between NSVT and HF hospitalization. They were relatively old and mainly hospitalized for HFpEF. In a previous study, patients with HFpEF had a relatively high NSVT burden and high risk of mortality.^14^ Therefore, progression to HFpEF should be noted in patients with NSVT without structural heart disease. Considering that the patients with NSVT in our population were older and had a higher prevalence of hypertension and CKD, these backgrounds were consistent with those of patients who were vulnerable to HFpEF.^15^, ^16^ Patients with HFpEF have been reported to experience a relatively high and often under‐recognized burden of NSVT.^14^ In this study, 32.5% of patients with HFpEF diagnosis had one or more episodes of NSVT on a 14‐day ambulatory ECG monitor, and 44.7% of the patients with HFpEF had NSVT detected during cardiac pacemaker interrogations. NSVT can be a stealthy and underappreciated sign of developing HF, reflecting high left ventricular end‐diastolic pressure.^17^

Based on the echocardiogram findings, there was a trend toward a larger LV diameter and lower EF in both patients with PVC and patients with NSVT. Notably, right ventricular systolic pressure was higher only in patients in the NSVT group, and the mitral valve annular early filling tissue Doppler velocity was lower. These findings suggest a hidden cardiac diastolic dysfunction in patients with NSVT.

AF may have played a significant role in the timing of the development of HF events in our population. In this study, patients with NSVT had a higher prevalence of AF than those without, and AF rhythm at the time of HF hospitalization was observed in two paroxysmal AF patients (and one persistent AF patient. AF rhythm was also observed at the time of HF hospitalization). Therefore, in patients with NSVT without structural heart diseases, it is important to focus on the burden of AF and heart rate control during AF to prevent hospitalization for HF.

Another patient was later diagnosed with hypertrophic cardiomyopathy (Table 4). Furthermore, it has been reported that one in seven patients with frequent PVCs without apparently structural heart disease had myocardial abnormality detected on CMR, and these abnormalities were associated with adverse clinical outcomes.^18^ NSVT may indicate subclinical abnormalities in patients with structurally normal hearts. Considering that most patients in our population were diagnosed with apparently normal hearts using echocardiography, further examinations, including CMR, may be needed in some patients. It is important for patients with NSVT to undergo increased follow‐up and intensified cardiac evaluation to detect underlying structural heart diseases. In this study, patients who were later found to have structural heart disease were younger, while patients 1, 3, and 4 were older (Table 4). In other words, the search for structural heart disease should be intensified in younger patients, while the progression of HFpEF should be closely monitored in elderly patients.

We also examined the number of consecutive beats in NSVT and found that among the five cases of HF hospitalization, two cases involved NSVT episodes with only three consecutive beats (Table 4). Therefore, in patients without structural heart disease, NSVT should be carefully monitored, irrespective of the number of consecutive beats. When we focused on the timing of HF hospitalizations, we observed that events were concentrated within the first 6 months, suggesting that close monitoring during this early phase is warranted.

### 4.4 NSVT and embolic events

Previous research has established a link between ventricular arrhythmia and ischemic stroke.^19^ In the current study, embolic events were observed more frequently in patients with NSVT than in those without. The mechanism is stroke or thromboembolic events from AF, which were more frequently observed in patients with NSVT in our population. The association between NSVT and AF is not fully understood. In theory, retrograde ventriculoatrial conduction can occur with ventricular arrhythmia, acting like atrial ectopic beats. Therefore, there is a possibility that ventricular arrhythmia can increase the risk of AF through retrograde ventriculoatrial conduction.^20^ Although AF complications were not considered more common in PVC patients in the current study due to their therapeutic interventions and patients' young background, AF was more common in patients with NSVT, defined as a continuum of PVCs. In the multivariate model, AF was independently associated with embolic events, which is consistent with this mechanism. Thus, we hypothesized that the number of atrial fibrillation complications was higher in patients with NSVT, and therefore, embolic events were more frequent in these patients.

### 4.5 Clinical implication

Patients with NSVT without structural heart disease have a higher incidence of HF. When HF developed, most cases were HFpEF, while some were accompanied by AF. For patients with NSVT without apparent structural heart diseases, careful follow‐up and control of risk factors for HF (especially HFpEF) are warranted. In addition, NSVT may suggest subclinical abnormalities in patients without structural heart disease, and further examinations, including CMR, will be considered.

### 4.6 Study limitations

Our study had some limitations. First, our database was derived from a cohort of patients admitted to a single cardiovascular hospital. Consequently, compared to the general population, our patients were strictly controlled for cardiovascular risk factors. Therefore, adaptation to the general community population may be difficult. Second, although some patients fell outside the cutoff values on echocardiography, the study included patients who were not considered to have apparent structural heart disease based on initial evaluation. Third, we did not distinguish the origin of the PVCs or NSVT in this study. However, a simple distinction between the presence or absence of NSVT is useful in clinical situations. Future research is needed to determine whether interventions targeting PVCs and NSVT depending on its origin can reduce adverse events. This is a future task for our study.

## 5 CONCLUSIONS

In patients without overt structural heart diseases who visited a specialized cardiovascular hospital, the frequency of PVCs was not associated with clinical events. In contrast, the presence of NSVT was associated with a higher risk of HF hospitalization.

## FUNDING INFORMATION

The authors received no specific funding for this article.

## CONFLICT OF INTEREST STATEMENT

Dr. Suzuki has received lecture fees from Daiichi Sankyo and Bristol‐Myers Squibb. Dr. Yamashita has received research funding and/or lecture fees from Daiichi Sankyo, Bayer Yakuhin, Bristol‐Myers Squibb, Pfizer, Nippon Boehringer Ingelheim, Ono Pharmaceutical, and Toa Eiyo.

## ETHICS STATEMENT

The study protocol was approved by the Ethics Committee of the Cardiovascular Institute.

## PATIENT CONSENT STATEMENT

All participants gave written informed consent.

## PERMISSION TO REPRODUCE MATERIAL FROM OTHER SOURCES

This manuscript does not include reproducible materials from external sources.

## Supporting information


**Table S1.** Baseline echocardiogram parameters according to the number of baseline PVCs.
**Table S2**. Baseline echocardiogram parameters according to the existence of NSVT.

## Data Availability

Due to the nature of this research, the participants did not agree for their data to be shared publicly; therefore, supporting data are not available.
