# Supplementary material for: Association between ventricular arrhythmia (premature ventricular contractions burden and nonsustained ventricular tachycardia) and cardiovascular events in patients without structural heart disease
Source: J Arrhythm. 2024 Dec 15;41(1):e13203. doi: 10.1002/joa3.13203 (PMC11730726; doi:10.1002/joa3.13203)
Supplement: Supplementary file 1 — Table S1. Baseline echocardiogram parameters according to the number of baseline PVCs. Table S2. Baseline echocardiogram parameters according to the existence of NSVT. [file JOA3-41-e13203-s001.docx]

**Supplementary Table 1. Baseline echocardiogram parameters according to the number of baseline PVCs**

|  | Number of PVCs (total, n = 6332, 100.0%) | | | *P value* |
| --- | --- | --- | --- | --- |
|  | 0-999  (n = 5507) | 1000-9999  (n = 531) | 10000-  (n = 294) |  |
| IVST (mm) | 8.7 ± 1.7  (n = 5384) | 8.5 ± 1.8  (n = 516) | 8.5 ± 1.8  (n = 288) | 0.062 |
| PWT (mm) | 8.3 ± 1.4  (n = 5384) | 8.2 ± 1.4  (n = 516) | 8.2 ± 1.4  (n = 288) | 0.175 |
| LVDd (mm) | 45.7 ± 4.4  (n = 5384) | 46.8 ± 4.1  (n = 516) | 48.1 ± 4.4  (n = 288) | < 0.001 |
| LVDs (mm) | 28.7 ± 4.3  (n = 5384) | 29.9 ± 4.1  (n = 516) | 31.7 ± 4.5  (n = 288) | < 0.001 |
| LVEF (%) | 67.2 ± 9.4  (n = 5384) | 65.2 ± 7.5  (n = 516) | 62.4 ± 9.0  (n = 288) | < 0.001 |
| LAD (mm) | 34.3 ± 6.1  (n = 5384) | 34.6 ± 6.4  (n = 516) | 34.4 ± 6.2  (n = 288) | 0.513 |
| RVSP (mmHg) | 27.3 ± 6.4  (n = 4402) | 26.9 ± 5.1  (n = 409) | 27.7 ± 5.0  (n = 232) | 0.288 |
| E (cm/s) | 58.7 ± 18.5  (n = 5120) | 58.4 ± 19.8  (n = 484) | 56.0 ± 20.7  (n = 277) | 0.053 |
| A (cm/s) | 54.8 ± 17.8  (n = 4876) | 55.3 ± 18.2  (n = 471) | 53.6 ± 21.1  (n = 273) | 0.466 |
| E/A | 5.8 ± 159.2  (n = 4857) | 1.1 ± 0.6  (n = 464) | 1.1 ± 0.6  (n = 263) | 0.733 |
| E’ (cm/s) | 7.6 ± 2.7  (n = 3144) | 7.8 ± 4.4  (n = 307) | 7.2 ± 3.3  (n = 183) | 0.101 |
| E/E’ | 9.3 ± 3.3  (n = 3116) | 9.2 ± 3.2  (n = 300) | 8.9 ± 3.2  (n = 172) | 0.232 |

Abbreviations: A, atrial filling velocity; E, early diastolic filling velocity; e’, Mitral valve annular early filling tissue Doppler velocity; E/e’ ratio, the ratio of diastolic filling velocity divided by average e′; IVST, interventricular septal thickness; LAD, left atrium diameter; LVDd, left ventricular end-diastolic diameter; LVDs, left ventricular end-systolic diameter; LVEF, Left ventricular ejection fraction. RVSP, right ventricular systolic pressure; PWT, posterior wall thickness.

**Supplementary Table 2. Baseline echocardiogram parameters according to the existence of NSVT**

|  | NSVT (-)  (n = 5878) | NSVT (+)  (n = 454) | *P value* |
| --- | --- | --- | --- |
| IVST (mm) | 8.6 ± 1.7  (n = 5750) | 8.9 ± 1.8  (n = 438) | < 0.001 |
| PWT (mm) | 8.3 ± 1.4  (n = 5750) | 8.6 ± 1.4  (n = 438) | < 0.001 |
| LVDd (mm) | 45.8 ± 4.4  (n = 5750) | 47.4 ± 4.3  (n = 438) | < 0.001 |
| LVDs (mm) | 28.7 ± 4.3  (n = 5750) | 30.8 ± 4.7  (n = 438) | < 0.001 |
| LVEF (%) | 66.9 ± 7.3  (n = 5750) | 65.1 ± 22.7  (n = 438) | 0.089 |
| LAD (mm) | 34.2 ± 6.1  (n = 5750) | 36.1 ± 6.8  (n = 438) | < 0.001 |
| RVSP (mmHg) | 27.2 ± 6.2  (n = 4681) | 28.3 ± 6.0  (n = 362) | 0.001 |
| E (cm/s) | 58.7 ± 18.5  (n = 5488) | 56.7 ± 21.2  (n = 393) | 0.064 |
| A (cm/s) | 54.7 ± 17.8  (n = 5258) | 56.0 ± 20.8  (n = 362) | 0.231 |
| E/A | 5.4 ± 153.4  (n = 5234) | 1.5 ± 7.6  (n = 350) | 0.627 |
| E’ (cm/s) | 7.7 ± 2.9  (n = 3355) | 6.9 ± 2.8  (n = 279) | < 0.001 |
| E/E’ | 9.3 ± 3.3  (n = 3323) | 9.6 ± 3.5  (n = 265) | 0.065 |

Abbreviations: A, atrial filling velocity; E, early diastolic filling velocity; e’, Mitral valve annular early filling tissue Doppler velocity; E/e’ ratio, the ratio of diastolic filling velocity divided by average e′; HCM, hypertrophic cardiomyopathy; IVST, interventricular septal thickness; LAD, left atrium diameter; LVDd, left ventricular end-diastolic diameter; LVDs, left ventricular end-systolic diameter; LVEF, left ventricular ejection fraction; RVSP, right ventricular systolic pressure; PWT, posterior wall thickness.
